# Abnormal cardiovascular sympathetic and parasympathetic responses to physical and emotional stimuli in depersonalization disorder

**DOI:** 10.3389/fnins.2015.00089

**Published:** 2015-03-26

**Authors:** Andrew P. Owens, Anthony S. David, David A. Low, Christopher J. Mathias, Mauricio Sierra-Siegert

**Affiliations:** ^1^Autonomic and Neurovascular Medicine Unit, Institute of Neurology, Imperial College LondonLondon, UK; ^2^Autonomic Unit, Institute of Neurology, University College LondonLondon, UK; ^3^Section of Cognitive Neuropsychiatry, Institute of Psychiatry, King's College LondonLondon, UK; ^4^School of Sport and Exercise Sciences, Liverpool John Moores UniversityLiverpool, UK; ^5^Depersonalization Research Unit, Institute of Psychiatry, King's College LondonLondon, UK

**Keywords:** depersonalization, depersonalization disorder, autonomic nervous system, heart rate variability, orienting response, orienting reflex

## Abstract

**Background:**

Depersonalization disorder (DPD) is characterized by a subjective sense of unreality, disembodiment, emotional numbing and reduced psychogenic (sudomotor) sympathoexcitation.

**Aims:**

Three related experiments utilized escalating physical and emotional challenges in 14 DPD participants and 16 controls aimed to elucidate (i) whether the cardiovascular sympathetic (SNS) and parasympathetic (PNS) nervous systems are implicated in DPD pathophysiology and (ii) if possible, to determine whether the blunted sympathoexcitation in DPD is peripherally or centrally mediated.

**Method:**

Participants completed the Beck Anxiety Inventory (BAI), Dissociative Experience Scale (DES), and Cambridge Depersonalization Scale (CDS). Study I recorded heart rate (HR) and blood pressure (BP) during 5 min supine baseline, 3 min sustained handgrip (HG), 3 min cold pressor (CP) and 5 min 60° head-up tilt (HUT). In study II, HR, BP, and heart rate variability (HRV) were recorded during 5 min simultaneous 60° HUT and continuous presentation of unpleasant images (5 s per image). Study III examined HR and BP orienting responses (ORs) to simultaneous 60° HUT and pseudorandom presentation of unpleasant, neutral and pleasant images (5 s per image 3 min 25 s). OR data was grouped by image valence post hoc.

**Results:**

DPD BAI (*p* = 0.0004), DES (*p* = 0.0002), and CDS (*p* ≤ 0.0001) scores were higher than controls. The DPD group produced diminished diastolic BP (DBP) (*p* = 0.045) increases to HG. Other indices were comparable between groups. DPD participants produced diminished systolic BP (SBP) (*p* = 0.003) and DBP (*p* = 0.002) increases, but greater (*p* = 0.004) HR increases to CP. In study II, DPD high frequency HRV (HF-HRV)—indicating parasympathetic vagal activity–was reduced (*p* = 0.029). In study III, DPD DBP was higher throughout the 5 s duration of HUT/pseudorandom unpleasant image presentation (1 s, *p* = 0.002, 2 s *p* = 0.033, 3 s *p* = 0.001, 4 s *p* = 0.009, 5 s *p* = 0.029).

**Conclusions:**

Study I's BP pressor data supports previous findings of suppressed sympathoexcitation in DPD. The greater HR increases to CP, decreased HF-HRV in study II, and increased DBP during unpleasant ORs in study III implicates the SNS and PNS in DPD pathophysiology. These studies suggest the cardiovascular autonomic dysregulation in DPD is likely to be centrally-mediated.

## Introduction

Depersonalization disorder (DPD) is a subjective sense of unreality affecting the self. It is usually comprised of derealization (one's surroundings feel unreal), attenuated emotional experience, including affect, nociception and even homeostatic drives (e.g., thirst, disgust and hunger) (Sierra et al., 2005; Simeon et al., 2008), as well as feelings of disembodiment and detachment (Lee et al., 2012). DPD has been conceptualized as a defensive, emotionally-disengaging response that is implemented to accommodate threat deemed as beyond ones' control (Phillips et al., 2001; Sierra et al., 2012). Symptoms of DPD are common in healthy people while approximately 1–2% of the population suffer from for chronic DPD (Sierra and David, 2011). Typical onset is between 16 and 23 years, with greater symptom severity associated with younger onset of depersonalization (Baker et al., 2003). Chronic DPD is typically treatment resistant and in the majority, continuous with little or no fluctuation, and independent of affective and personality symptoms. Nevertheless symptoms can be exacerbated by stress, social anxiety and secondary to panic disorder (Simeon et al., 2003b).

DPD subjects produce decreased hypothalamic and amygdalae responses during presentation of happy and sad expressions respectively (Lemche et al., 2007, 2008), findings opposite to those seen in healthy controls. Levels of noradrenaline (NA), a key central autonomic neurotransmitter, have been shown to be negatively correlated with DPD severity (Simeon et al., 2003a). DPD patients share some symptoms with patients with corticolimbic disconnections (Mayer-Gross, 1935; Sierra et al., 2002a), supporting the hypothesis that, in DPD, the expected vs. the actual experience of emotions are discrepant, leading to the experience of emotional numbing and disembodiment.

Skin conductance responses (SCRs) provide an index of sudomotor sympathetic nervous system (SNS) activity. Models of DPD predict hypervigilance of environmental and emotional stimuli and the engagement of an emotionally dampening mechanism during emotional aversion, as evidenced by reduced SCRs to unpleasant images compared to both healthy controls and anxiety disorder patients, despite DPD subjects being equally as anxious as anxiety participants (Sierra et al., 2002b). SCRs are both more quickly manifested and yet abnormally weakened in DPD participants during emotionally aversive stimuli exposure (Sierra et al., 2002b), indicating hypervigilant attentional appraisal and subsequent rapid suppression.

In DPD, an abnormally strong interaction between right ventral prefrontal cortex (PFC) and left insular responses during unpleasant emotional stimuli—normally inversely related (Tabibnia et al., 2008)—has been reported (Phillips et al., 2001), as well as between happy and sad expression intensity and right hypothalamus and right amygdala responses respectively. Central autonomic networks within the spinal cord, brainstem, and hypothalamus are responsible for controlling cardiovascular and thermoregulatory autonomic outflows and haemodynamic and sudomotor changes are global autonomic responses that involve the cortex, limbic forebrain and midbrain (Westerhaus and Loewy, 2001; Saper, 2002). In studies utilizing simultaneous functional neuroimaging and SCR measurements, inverse correlations between SCRs and dorsal PFC responses have also been described in DPD (Lemche et al., 2007, 2008), however, the direction of the relationship between central and peripheral findings remains unclear. Moreover, autonomic investigations of DPD have predominantly focused on electrodermal activity as an index of sudomotor sympathetic autonomic function, neglecting analysis of the cardiovascular SNS and parasympathetic nervous systems (PNS).

Considering these findings that have investigated central (via neuroimaging) and sudomotor SNS responses (via electrodermal activity) in DPD, in the current study, we have utilized a series of physical and emotional challenges in a group of DPD participants and healthy controls using validated clinical autonomic investigations (Mathias et al., 2013) and emotional stimuli (Lang et al., 2005) to determine (i) whether the cardiovascular SNS and PNS are also implicated in DPD pathophysiology and (ii) whether the blunted sympathoexcitation in DPD is peripherally or centrally mediated.

## Methods

### Sample

All experimental procedures were ethically approved by Imperial College Healthcare Trust Research and Design Office, South London and Maudsley NHS Foundation Trust ethics committees and the North West London REC 2 NRES Committee. The study was conducted in compliance with the Helsinki declaration (World Medical Association, 1994). Thirty participants (22 men and 8 women) were tested, 16 healthy controls (mean age 33.36 ± 9.97 years, mean educational level 2.88, where 1 = primary, 2 = secondary, and 3 = higher) and 14 DPD patients (mean age 30.5 ± 9.83 years, mean educational level 2.93) with a DSM-IV diagnosis of DPD were recruited from the Depersonalization Disorder Clinic, Maudsley Hospital.

All patients were diagnosed with chronic and continuous (as opposed to intermittent) DPD. The diagnosis of DPD was ascertained by means of a semi-structured interview using the Present State Examination (Wing et al., 1974) and by scores above the cut-off point of 70 on the Cambridge Depersonalization Scale (CDS) (Sierra and Berrios, 2000). Written informed consent was provided by all participants prior to participation. Autonomic testing was carried at the Autonomic and Neurovascular Medicine Unit, St Mary's Hospital (Imperial College Healthcare Trust), a national referral center for cardiovascular and sudomotor dysautonomia using the London Clinical Neurosciences (LoCAN) Group protocol (Mathias et al., 2013). Exclusion criteria included any history of condition likely to effect cardiovascular function, such as peripheral neuropathy or diabetes mellitus. Controls were also required to not have a history of an affective or dissociative disorder.

### Self-report measures

In addition to the CDS (Sierra and Berrios, 2000), dimensions for dissociation and anxiety were taken using the Dissociative Experience Scale (DES) (Bernstein and Putnam, 1986), and the Beck Anxiety Inventory (BAI) (Beck et al., 1988), respectively.

### Study I: cardiovascular autonomic function tests

Study I aimed to investigate the cardiovascular sympathetic nervous system using physical stimuli. Ambient temperature of the treatment room was maintained at 21°C throughout testing for all participants and hear rate (HR) and heart rate variability (HRV) were recorded using the PowerLab 16/30/ECG (Bioamp) (AD Instruments, Oxford, United Kingdom) and analyzed using the Labchart 7 software package for the three experiments. Blood pressure (BP) was continually recorded using Finometer (Smart Medical, Gloucestershire, United Kingdom) and intermittent BP and HR measures were taken using Dinamap Pro400V2 (GE Healthcare, Buckinghamshire, United Kingdom).

Participants lay in the supine position for 5 min to establish a baseline recording of systolic BP (SBP), diastolic BP (DBP), HRV, and HR. Pressor exercises examine cardiovascular SNS integrity during physiological provocation and have been well-validated (Khurana and Setty, 1996; Mathias, 2003; Mathias et al., 2013). Pressor maneuvers, including isometric (hand-grip) exercise, cutaneous cold application and mental arithmetic provide an index of sympathetic nerve activity (SNA) and induce autonomic changes, particularly BP, which is regulated via the SNS. Isometric and cutaneous cold pressor stimuli raise BP via activation of sympathetic efferent nerve pathways and provide the most responsive data in comparison to mental arithmetic or other pressor tests. Peripheral receptors are activated during pressor maneuvers but in both cutaneous cold or isometric exercise tests there is a central command (isometric) or nociceptive (cold) role, which is more pronounced in isometric exercise study leading to a greater increase SNA in this test compared to the cold pressor. Pressor exercises were carried out in the supine position, so that orthostatic demand does not confound the pressor responses.

During isometric handgrip exercise (HG), the participants were requested to sustain a handgrip at 1/3 of maximum voluntary contraction pressure for 3 min using a gauge directly in front of them. After the isometric exercise had been completed, a second baseline of a minimum of 3 min elapsed allowing autonomic activity to return to baseline levels before the cutaneous cold pressor (CP) exercise was carried out. The subjects' right hand was placed in an icepack chilled to 4°C for 3 min, tolerance permitting. Cardiovascular autonomic activity was then allowed to return to baseline levels (minimum of 3 min) before 5 min 60° head-up tilt (HUT). HUT is used to diagnose various forms of dysautonomia. In healthy subjects, the initial BP fall induced by HUT should recover within 60 s because when decreased venous return to the heart causes reduced stroke volume and cardiac output, arterial baroreceptors and cardiopulmonary mechanoreceptors then signal autonomic brain centers to increases SNA, raising HR and causing vasoconstriction of the blood vessels in various vascular beds to compensate for postural and gravitational demands (Imholz et al., 1990; Mathias et al., 2013). In normal subjects where the baroreflex is intact, HUT of 45–90° should not provoke a prolonged fall in BP.

### Study II: heart rate variability during simultaneous HUT and anticipated unpleasant images

Study II examined cardiovascular sympathetic and parasympathetic responses to simultaneous anticipated and sustained physical and emotional stimuli. Ambient temperature of the treatment room was maintained at 21°C throughout testing for all participants. HR, BP, and HRV were continuously recorded. Participants lay in the supine position for 5 min to establish a baseline recording of SBP, DBP, HRV, and HR. After 5 min 60° HUT baseline and whilst still on tilt, subjects were presented with a constant stream of 60 unpleasant images from the International Affective Picture System (IAPS) for 5 min (5 s per slide) from a screen 12 inches from the subject's face. The IAPS is a database of images of varying quantified valences (neutral, pleasant, and unpleasant) used to investigate emotional processing (Lang et al., 2005). Visual emotional stimuli share many perceptual and sensory aspects to the object they depict and so effectively activate motivational networks and, in this case, provided measure of sympathetic and parasympathetic responses to a sustained and anticipated emotional stressor previously found to produce functional neuroanatomical differences in cognitive-affective processing in DPD.

During simultaneous HUT and unpleasant images, the lights in the treatment room were turned off to assist the participant in maintaining their attention on the monitor screen. Spectral analytical techniques of short or long-term cardiovascular changes provide a measure of cardiovagal activity, with the two main spectral components being defined as high frequency [HF (0.15–0.4 Hz)], which predominantly depicts vagal influences and is comparable to respiratory sinus arrhythmia (RSA), and low frequency [LF(0.04–0.15 Hz)].

LF heart rate variability (HRV) was, until recently, believed to depict sympathetic cardiac influences (Malliani et al., 1991) however, LF HRV as a purely sympathetic measure has been called into question (Parati et al., 2006; Goldstein et al., 2011) as research has shown that endogenous fluctuations in LF HRV provide information about sympathetic regulation of BP, such as vasomotor tone and baroreceptor activity. Moreover, recent studies have positively correlated LF HRV and baroreceptor sensitivity (Moak et al., 2007; Goldstein et al., 2011) as well as reduced LF HRV and baroreflex-cardiovagal failure (Rahman et al., 2011). Therefore, LF HRV may well provide information about sympathetic mechanisms but perhaps not cardiac SNA specifically but rather of baroreflex function and dysfunction.

The participants' HRV was evaluated post hoc using the Fast Fourier Transformation (FFT) model of spectral analysis. RR intervals of each participant were transformed into bands with different spectral frequencies. HRV during isometric exercise and cutaneous cold pressor maneuvers was not analyzed due to the minimum required duration for analysis being 5 min (Task Force of the European Society of Cardiology and the North American Society of Pacing and Electrophysiology, 1996) and these exercises only lasting 3 min and spectral analysis requiring relatively stable cardiac activity.

### Study III: orienting responses to simultaneous HUT and mixed valence images

Study III examined cardiovascular autonomic orienting responses to pseudorandomized emotional stimuli of neutral, pleasant and unpleasant valences. As before, ambient temperature of the treatment room was maintained at 21°C throughout testing. HR and BP were continuously recorded. Participants lay in the supine position for 5 min to establish a baseline recording of SBP, DBP, and HR.

The orienting response (OR) is a spectrum of transient physiological and behavioral adjustments, typified by increased parasympathetic tone, such as bradycardia or reduced sympathetic tone, weakened SCRs, elicited by the occurrence of a novel stimulus. This “investigatory reaction” was first described by Pavlov in animal studies as a behavioral adjustment of faculties to the novel cue (Pavlov, 1927). It is proposed that the physiological downregulation during ORs facilitates cognitive processing and appropriate behavioral response to the stimulus (Turpin, 1986). After 5 min 60° HUT baseline, the subjects were pseudorandomly presented with 14 neutral images, 13 unpleasant images, and 12 pleasant images (5 s per image, total of 3 min 25 s) on a monitor 12 inches from the participant's face. As before, the lights in the treatment room were turned off to assist the participant in maintaining their attention on the screen.

### Statistical analysis

Self-report questionnaire data, baseline, clinical and experimental outcome variables between DPD and control participants were analyzed. For all analyses, the null hypothesis was evaluated at a two-sided significance level of 0.05, with calculation of 95% confidence intervals. Nonparametric statistical methods were used throughout (Mann-Whitney *U*-test) given that questionnaire and cardiovascular outcome variables within the DPD cohort had a skewed distribution. All data analysis was carried out using SPSS version 17.

## Results

### Self-report measures

The DPD cohort scored significantly higher on all three self-report instruments of anxiety (BAI, *p* = 0.0004), dissociation (DES, *p* = 0.0002) and depersonalization (CDS, *p* = 0.0001) in comparison to normal controls, as expected (see Table 1). One healthy control failed to complete the CDS and DES.

**Table 1:** **Mean questionnaire scores, including standard deviation and *p*-values**.

**Questionnaires**	**Groups**	**Mean Scores**	***P*-Value**
Cambridge Depersonalization Scale	DPs	141.79 ± 37.5^***^	< 0.0001
	Controls	19.33 ± 15	
Beck anxiety inventory	DPs	22.14 ± 11.3^***^	
	Controls	9.88 + 6.7	0.0004
Dissociative Experiences Scale	DPs	709.29 + 416.6^***^	
	Controls	188.67 ± 88.1	0.0002

*** *P < 0.001*.

### Study I: cardiovascular autonomic function tests

Cardiovascular autonomic responses to physical stimuli (HG, CP, HUT) were present in all clinical and control participants, none of whom provided evidence of cardiovascular autonomic failure (AF) or peripheral neuropathy. During 5 min supine baseline, both groups produced similar cardiovascular profiles. During 3 min HG, HR, SBP, and DBP increased in both cohorts but DBP (*p* = 0.045) increases in the DPD group were significantly blunted compared to controls (see Table 1). DPD participants also produced blunted SBP (*p* = 0.003) and DBP (*p* = 0.002) increases to cutaneous CP but greater (*p* = 0.004) HR increases in comparison to controls during CP (see Table 2). One DPD participant could only tolerate the cold pressor exercise for 2 min. During 5 min HUT, both groups produced comparable autonomic profiles.

**Table 2:** **Mean HR and BP responses during pressor exercises in controls and DPDs**.

	**HR Δ**	**SBP Δ**	**DBP Δ**
**ISOMETRIC HAND GRIP EXERCISE**			
Controls	3.07 ± 10.57	19.23 ± 20.54	16.39 ± 13.51
Depersonalization group	2.93 ± 5.22	15.85 ± 17.44	3.25 ± 11.58^*^
**CUTANEOUS COLD PRESSOR**			
Controls	−2.00 ± 3.40	11.88 ± 16.14	10.54 ± 19.14
Depersonalization group	4.21 ± 6.84^*^	−12.32 ± 19.86^*^	−8.28 ± 12.41^*^

*HR, heart rate; SBP, systolic blood pressure; DBP, diastolic blood pressure*.

* *P < 0.05*.

### Study II: heart rate variability during simultaneous HUT and anticipated unpleasant images

**Figure 1:**
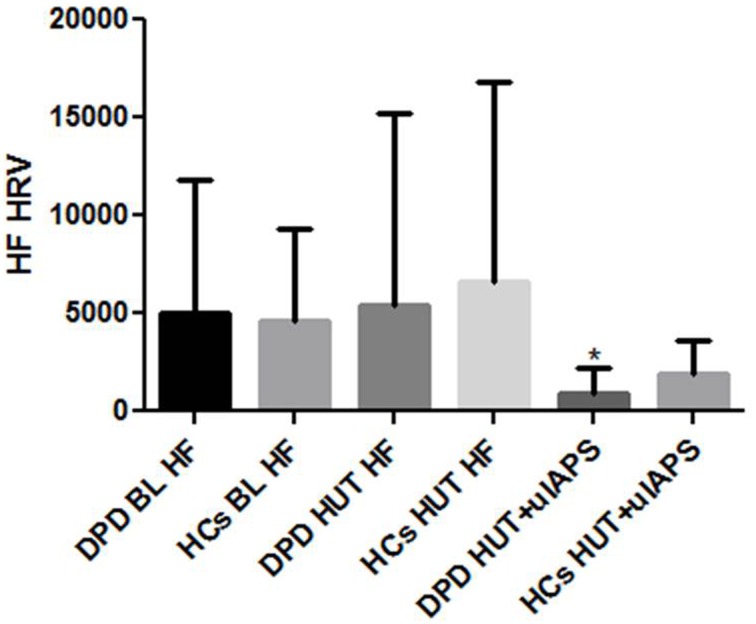
**HF-HRV data during supine baseline (BL), head up tilt (HUT), and HUT viewing of unpleasant images**. DPD, Depersonalization Disorder group; HC, healthy control group; uIAPS, unpleasant images from the International Affective Picture System. ^*^P ≤ 0.05.

During 5 min supine baseline and HUT baseline, both groups produced similar HRV and BP profiles. LF changes during 5 min simultaneous HUT and unpleasant images were not significant between groups, however, there was a significantly greater reduction (*p* = 0.029) in HF-HRV in the DPD group in comparison to controls (see Figure 1). BP responses to HUT did not differ significantly between groups.

### Study III: orienting responses to simultaneous HUT and mixed valence images

**Figure 2:**
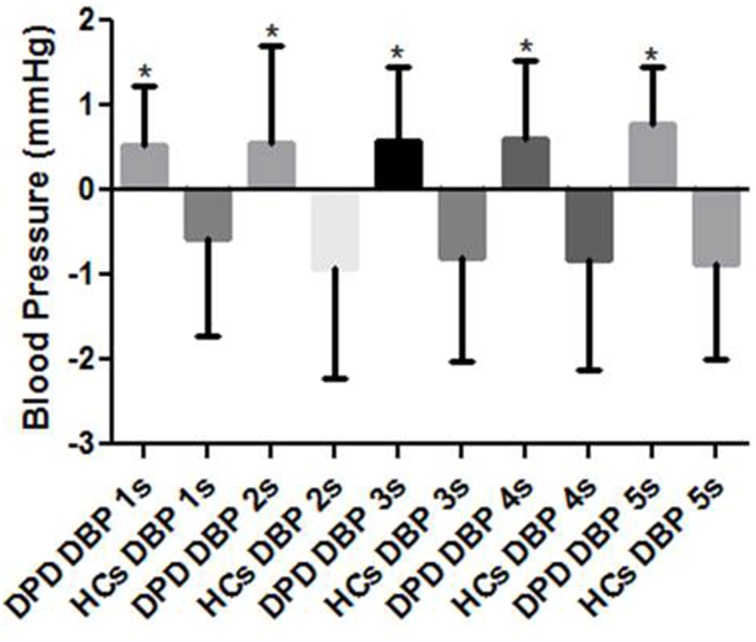
**Mean DBP responses to simultaneous HUT and pseudorandom unpleasant images**. DPD, Depersonalization Disorder group; HC, Healthy Control group; DBP, diastolic blood pressure. ^*^P < 0.05.

Though images were presented in a pseudorandom order, response data were grouped into valence categories of neutral, pleasant and unpleasant post hoc. There were no group differences in cardiac or SBP ORs, however, throughout the 5 s presentation of HUT and unpleasant images, the DPD group produced an increase rather than decrease in DBP at 1 s (*p* = 0.002), 2 s (*p* = 0.033), 3 s (*p* = 0.001), 4 s (*p* = 0.009), and 5 s (*p* = 0.029) (see Figure 2).

## Discussion

This study used three related experiments involving escalating physical (Mathias et al., 2013) and emotional challenges (Lang et al., 2005) in a group of DPD participants and healthy controls to examine (i) whether the cardiovascular SNS and PNS are also implicated in DPD pathophysiology and (ii) whether the blunted sympathoexcitation in DPD is peripherally or centrally mediated.

In study I, there was no evidence of autonomic failure (AF), orthostatic intolerance (OI) or any other symptoms (organic or reported) during physical stimuli (except for one DPD participant who could only tolerate 2 min of CP), however, there was a blunted DBP (*p* = 0.045) response to HG in the DPD group, and blunted SBP (*p* = 0.003), and DBP (*p* = 0.002) responses to CP in this group also. The DPD cohort also produced a greater HR rise (*p* = 0.004) to CP in comparison to the control group, which may be associated with the reduced vagal tone (HF-HRV) evidenced in study II. In study II, HF-HRV during HUT and unpleasant image presentation was reduced in the DPD group (*p* = 0.029). In study III, DPD participants produced increases rather than decreases in DBP during ORs throughout the 5 s epoch of simultaneous HUT/unpleasant images (1 s, *p* = 0.002, 2 s *p* = 0.033, 3 s *p* = 0.001, 4 s *p* = 0.009, 5 s *p* = 0.029).

### Study I discussion: cardiovascular autonomic function tests

In comparison to the CP, which itself has a nociceptive component and may explain the current HR increases in the DPD group during this exercise (Kalisch et al., 2005; Pollatos et al., 2012), the increased SNA during HG is exacerbated by the recruitment of central command to maintain handgrip in addition to the utilization of ergoreceptors and chemoreceptors (Mathias et al., 2013). The HG and CP findings in study I are unlikely to result from peripheral vasodilation or reduced oxygen delivery to peripheral tissue due to none of the control or DPD participants presenting any evidence of autonomic failure (AF) or peripheral neuropathy during supine or HUT baselines and pressor responses being present in both groups, despite the blunted DBP increase in DPD participants. DBP dysregulation has previously been demonstrated, for example, post-exercise CP and mental arithmetic DBP responses were found to be blunted in sedentary males and females (West et al., 1998) and bi-directional DBP responses can also be conditioned in humans (Elder et al., 1979).

The nucleus of the solitary tract (NTS) receives baroreceptor afferents that synapse with the rostral ventrolateral medulla to set efferent pressor tone. Reduced baroreceptor tone and initiation of baroreflexes are associated with anterior cingulate cortex (Acc), amygdala, PFC and insula function, (Kimmerly et al., 2005), areas also implicated in DPD neuropathophysiology SBP indicates stroke volume, aortic compliance and left ventricular ejection velocity, whereas DBP indicates peripheral resistance of blood flow from arterioles to capillaries and is chiefly dependent on cardiac output. Behavior-dependent increases in BP are both enabled and moderated by the baroreflex, whereas the cutaneous circulation is predominantly regulated through the rostral ventromedial medulla, rostral ventrolateral medulla and medullary raphe (Dampney et al., 2002).

The HG and CP findings in study I are unlikely to result from peripheral vasodilation or reduced oxygen delivery to peripheral tissue due to none of the control or DPD participants presenting any evidence of autonomic failure (AF) or peripheral neuropathy during supine or HUT baselines and pressor responses being present in both groups, despite the blunted DBP increase in DPD participants. DBP dysregulation has previously been demonstrated, for example, post-exercise CP and mental arithmetic DBP responses were found to be blunted in sedentary males and females (West et al., 1998) and bi-directional DBP responses can also be conditioned in humans (Elder et al., 1979).

Long-term regulation of arterial pressure is not mediated by changes in peripheral resistance, which may help explain why the otherwise normal BP profile of the DPD group was dysregulated during study I's pressor exercises and accompanying central stressors, such as nociception (CP) and central command (HG). The pressor findings provide further evidence of the disrupting effects of DPD on sympathoexcitation, though the normal responses in the DPD group to baseline HUT suggests that this blunted sympathoexcitation may be exacerbated by the recruitment of central processes during HG and CP. The greater HR increase to the CP in the DPD group may be further evidence of the attenuated parasympathetic vagal tone evidenced by the decreased HF-HRV during simultaneous physical and emotional stimuli in study II.

Brain regions thought to function aberrantly during emotional processing in DPD are engaged during pressor exercises (Fish et al., 1993; Harper et al., 2000; Critchley et al., 2000a, 2005b; Dalton et al., 2005; Lemche et al., 2007, 2008; Sierra and David, 2011). Increased activity in the medial PFC, anterior and posterior insular, and ventroposterior thalamus during HG has been reported in healthy controls (King et al., 1999), as well as increased activation in the anterior cingulate cortex, insula, medial temporal lobe, ventral and medial PFC, medial thalamus, cerebellum midbrain and pons during CP in healthy controls (Harper et al., 2000). Critchley et al. (2000a) assessed brain activity during HG and mental arithmetic exercises in healthy controls, finding that increases in BP were positively correlated with anterior cingulate activity.

The only study of structural imaging in DPD to date has shown reduced cortical thickness in the right middle temporal region and some changes in inferior frontal regions (Sierra et al., 2014). Functional neuroimaging of DPD participants has also provided neuroanatomical correlates for the emotional numbing that typifies depersonalization, demonstrating abnormal interactions between right ventral PFC activity and left insular responses during unpleasant emotional stimuli in comparison to healthy controls (Phillips et al., 2001), and between happy and sad expression intensity and right hypothalamus and right amygdala responses respectively. The implication of the right ventral PFC and left insular in DPD neuropathophysiology are particularly relevant to this study and autonomic function in general as sympathetic responses are lateralized to the right hemisphere (Oppenheimer et al., 1992) with the left insular cortex involved in parasympathetic cardiovascular regulation, for example, acute left insular stroke disrupts the correlation between HR and BP (Oppenheimer et al., 1996). The implication of limbic dysregulation modulated by PFC emotional reappraisal in the neuropathophysiology of DPD may have contributed to the current findings during pressor exercises and their additional central demands.

### Study II discussion: heart rate variability during simultaneous HUT and anticipated unpleasant images

During the simultaneous HUT and constant stream of unpleasant images in study II, DPD subjects produced a significant decrease in HF-HRV, indicating reduced parasympathetic vagal. The SNS and PNS often work antagonistically and with organ specificity, producing definable patterns of autonomic activity, including functionally and emotionally-specific autonomic patterns (Ekman et al., 1983; Rainville et al., 2006). Disruption of complex emotional responses, such as empathy, can occur in central or peripheral autonomic lesion deficit models, such as pure autonomic failure (PAF) (Heims et al., 2006; Chauhan et al., 2008), multiple system atrophy (MSA) (Kaye et al., 2006; Brown et al., 2010) and spinal cord injury (SCI) (Nicotra et al., 2006), indicating the emotional impairment of insufficient reciprocal autonomic arousal.

HR changes are predicted by amygdala and dorsal Acc activity (Janig and Habler, 2003) and during threat/stress induction, amygdala function predicts cardiac contractility (Dalton et al., 2005). The amygdala and limbic cortices supply a descending efferent drive to the hypothalamus and brainstem for congruent autonomic responses to emotion-related behavior (Saper, 2002). Dorsal anterior cingulate cortex (Critchley et al., 2003) and insula cortex (Critchley et al., 2000a,b) activity reflects the engagement of sympathetic activity coupled to mental and physical behaviors. The right anterior insula and left inferior anterior insula have been identified as neural correlates of alexithymia (difficulty in experiencing and expressing emotion) in DPD (Lemche et al., 2013). Both the insula and cingulofrontal areas are important for emotional perception (Critchley et al., 2001; Katkin et al., 2001) and the anterior insula is implicated in empathy due to its role in homeostatic modifications that integrate interoceptive and autonomic processes.

The HF-HRV data in study II appears to contradict previous research findings of decreased sympathoexcitation to aversive stimuli in depersonalized individuals (Lemche et al., 2007, 2008). However, previous studies have predominantly focused on sudomotor SNS activity as an index of “autonomic” function, neglecting analysis of the cardiovascular SNS and PNS. Cardiovascular autonomic investigations provide a more comprehensive insight into the function and integrity of the subject's autonomic nervous system (ANS). Moreover, one recent study concluded that DPD patients have greater SCR ranges rather than smaller skin conductance levels (Lemche et al., 2008), and the only other study to have used HRV in DPD subjects (Schoenberg et al., 2012) found reduced LF *and* HF during sham biofeedback conditions, supporting what the current data indicate, that both sympathetic and parasympathetic nervous systems are dysregulated in DPD. The reduced activity of cardioinhibitory vagal fibers emanating from the nucleus ambiguous (Neff et al., 2003) evidenced by the reduced HF-HRV may also explain the exaggerated HR increase to the CP in the DPD group in study I.

### Study III discussion: orienting responses to simultaneous HUT and mixed valence images

During simultaneous HUT/mixed valence image presentation, DPD participants produced significantly increased DBP responses during unpleasant image ORs, yet the expected simultaneous reduction in SBP and HR during ORs in the DPD cohort was comparable to controls and typical of normal ORs (Turpin, 1986). Short-term arterial pressure fluctuations are autonomically-mediated by factors, such as, barosensitive sympathetic efferent neurons that innervate kidney and cardiac tissue and mediate noradrenaline (NA) release from adrenal chromaffin cells that constrict non-dermal resistance arterioles. BP control involves a number of neurohormones, such as antidiuretic hormone (ADH), NA, adrenaline and the renin-angiotensin system (RAS) that bring about vasoconstriction and water retention.

NA and angiotensin II have a positive postsynaptic interaction with SBP but not DBP (Struthers et al., 1987) which may account for the bi-directional DBP activity in the DPD group. The negative correlation between DPD severity and NA (Simeon et al., 2003a) and the PFC and limbic centers implicated in DPD emotional processing may influence the SNA of one or more autonomic-mediated systems.

Throughout vigilant attention, Acc metabolic activity is suppressed by increased middle PFC activation (Lewin et al., 1996) and projections from the Acc and medial temporal lobe congregate within the ventromedial PFC, an area involved in autonomic control and emotional appraisal (Ongur et al., 2003). Amygdala activity predicts the inflection of anxiety-related cardiac responses (Janig and Habler, 2003; Critchley et al., 2005b), as well as predicting emotion-induced changes in dorsal anterior cingulate cortex activity and cardiac response (Critchley et al., 2005a). Therefore, from a top-down perspective, the current OR findings could be due to limbic/autonomic centers, such as the dorsomedial hypothalamus (environmental cardiovascular responses (Dampney et al., 2002), paraventricular neucleus of the hypothalamus [afferent homeostatic axis (Benarroch, 2005)] or amygdala [sympathoadrenal responses (Janig and Habler, 2003)] that function aberrantly during aversive emotional processing in DPD (Phillips et al., 2001; Medford et al., 2006; Lemche et al., 2007, 2008) dysregulating downstream effector organ responses.

Aberrant PFC suppression of subcortical function has previously been proposed as a neuroanatomical basis of DPD symptomatology (Medford et al., 2006; Lemche et al., 2007, 2008) and may have been exacerbated during ORs to simultaneous HUT/pseudorandom unpleasant image presentation in study III, pressor responses (study I) and simultaneous HUT/constant unpleasant image presentation (study II) by the combination of attentional, affective and physiological stressors.

### Limitations

This study had some limitations, the most significant being the small sample size, which can exacerbate high degrees of variance within the sample as the physical stimuli that were used to test SNS activity measured effector organ activity; the most direct would be sympathetic microneurography, which is an invasive technique and would have been impractical and potentially distressing for the participants, making baseline recordings difficult. There also are no direct measures in humans to assess dynamic cardiac parasympathetic activity, hence the use of techniques such as HF-HRV. Another issue is the difficulty in teasing out effects of anxiety/low mood from the core features of DPD. Future studies of DPD neurophysiology should consider including an anxiety control group.

### Implications

This research set out to elucidate further the autonomic pathophysiology of DPD, specifically (i) whether the cardiovascular sympathetic (SNS) and parasympathetic (PNS) nervous systems are also implicated in DPD pathophysiology and (ii) if possible, to determine whether the blunted sympathoexcitation in DPD is peripherally or centrally mediated. The blunted pressor BP profiles in study I support previous findings of suppressed sympathoexcitation in DPD, however, the greater HR increase during CP—which has a nociceptive (emotional) component, the decreased HF-HRV during HUT/unpleasant images in study II, and increased DBP during ORs in study III indicates that both the PNS *and* SNS function is implicated in the pathophysiology of DPD, rather than sudomotor sympathetic suppression alone. This dysregulation is unlikely to be peripherally-mediated due to normal supine baseline and HUT baseline findings and present pressor responses in both groups of participants, in none of whom was there evidence of a peripheral neuropathy or autonomic failure. Impaired autonomic responses may provide a basis for some of the core subjective disturbances reported by DPD patients. Future treatment strategies for DPD may benefit from the inclusion of combined pharmacological and non-drug therapy, the latter including biofeedback with a view to increasing appropriate autonomic arousal during physical and emotional stimuli.

### Conflict of interest statement

The authors declare that the research was conducted in the absence of any commercial or financial relationships that could be construed as a potential conflict of interest.

## Abbreviations

Acc: anterior cingulate cortex
ADH: antidiuretic hormone
AF: autonomic failure
ANS: autonomic nervous system
BAI: Beck anxiety inventory
BP: blood pressure
CDS: Cambridge depersonalization scale
CP: cold pressure
DES: Dissociative experiences
DBP: diastolic blood pressure
DPD: depersonalization disorder
HF: high frequency heart rate variability
HG: isometric hand grip exercise
HR: heart rate
HRV: heart rate variability
HUT: head up tilt
IAPS: International Affective Picture System
LF: low frequency heart rate variability
MSA: multiple system atrophy
NA: noradrenaline
NTS: nucleus of the solitary tract
PAF: pure autonomic failure
PFC: prefrontal cortex
PNS: parasympathetic nervous system
RAS: renin-angiotensin system
SBP: systolic blood pressure
SCI: spinal cord injury
SNA: sympathetic nerve activity
SNS: sympathetic nervous system.

## References

[B1] Baker D. Hunter E. Lawrence E. Medford N. Patel M. Senior C.. (2003). Depersonalisation disorder: clinical features of 204 cases. Br. J. Psychiatry 182, 428–433. 10.1192/bjp.182.5.428 12724246

[B2] Beck A. T. Epstein N. Brown G. Steer R. A. (1988). An inventory for measuring clinical anxiety: psychometric properties. J. Consult. Clin. Psychol. 56, 893–897. 10.1037/0022-006X.56.6.893 3204199

[B3] Benarroch E. E. (2005). Paraventricular nucleus, stress response, and cardiovascular disease. Clin. Auton. Res. 15, 254–263. 10.1007/s10286-005-0290-7 16032381

[B4] Bernstein E. M. Putnam F. W. (1986). Development, reliability, and validity of a dissociation scale. J. Nerv. Ment. Dis. 174, 727–735. 10.1097/00005053-198612000-00004 3783140

[B5] Brown R. G. Lacomblez L. Landwehrmeyer B. G. Bak T. Uttner I. Dubois B.. (2010). Cognitive impairment in patients with multiple system atrophy and progressive supranuclear palsy. Brain 133, 2382–2393. 10.1093/brain/awq158 20576697

[B6] Chauhan B. Mathias C. J. Critchley H. D. (2008). Autonomic contributions to empathy: evidence from patients with primary autonomic failure. Auton. Neurosci. 140, 96–100. 10.1016/j.autneu.2008.03.005 18442952

[B7] Critchley H. D. Corfield D. R. Chandler M. P. Mathias C. J. Dolan R. J. (2000a). Cerebral correlates of autonomic cardiovascular arousal: a functional neuroimaging investigation in humans. J. Physiol. 523(Pt 1), 259–270. 10.1111/j.1469-7793.2000.t01-1-00259.x 10673560 PMC2269796

[B8] Critchley H. D. Elliott R. Mathias C. J. Dolan R. J. (2000b). Neural activity relating to generation and representation of galvanic skin conductance responses: a functional magnetic resonance imaging study. J. Neurosci. 20, 3033–3040. 10751455 10.1523/JNEUROSCI.20-08-03033.2000 PMC6772223

[B9] Critchley H. D. Mathias C. J. Dolan R. J. (2001). Neuroanatomical basis for first- and second-order representations of bodily states. Nat. Neurosci. 4, 207–212. 10.1038/84048 11175883

[B10] Critchley H. D. Mathias C. J. Josephs O. Oídoherty J. Zanini S. Dewar B. K.. (2003). Human cingulate cortex and autonomic control: converging neuroimaging and clinical evidence. Brain 126:2139. 10.1093/brain/awg216 12821513

[B11] Critchley H. D. Rotshtein P. Nagai Y. O'Doherty J. Mathias C. J. Dolan R. J.. (2005a). Activity in the human brain predicting differential heart rate responses to emotional facial expressions. Neuroimage 24, 751–762. 10.1016/j.neuroimage.2004.10.013 15652310

[B12] Critchley H. D. Tang J. Glaser D. Butterworth B. Dolan R. J. (2005b). Anterior cingulate activity during error and autonomic response. Neuroimage 27, 885–895. 10.1016/j.neuroimage.2005.05.047 15996878

[B13] Dalton K. M. Kalin N. H. Grist T. M. Davidson R. J. (2005). Neural-cardiac coupling in threat-evoked anxiety. J. Cogn. Neurosci. 17, 969–980. 10.1162/0898929054021094 15969913

[B14] Dampney R. A. Coleman M. J. Fontes M. A. Hirooka Y. Horiuchi J. Li Y. W.. (2002). Central mechanisms underlying short- and long-term regulation of the cardiovascular system. Clin. Exp. Pharmacol. Physiol. 29, 261–268. 10.1046/j.1440-1681.2002.03640.x 11985533

[B15] Ekman P. Levenson R. W. Friesen W. V. (1983). Autonomic nervous system activity distinguishes among emotions. Science 221, 1208–1210. 10.1126/science.6612338 6612338

[B16] Elder S. T. Gamble E. H. McAfee R. D. van Veen W. J. (1979). Conditioned diastolic blood pressure. Physiol. Behav. 23, 875–880. 10.1016/0031-9384(79)90194-X 523543

[B17] Fish D. R. Gloor P. Quesney F. L. Olivier A. (1993). Clinical-Responses to Electrical Brain-Stimulation of the temporal and frontal lobes in patients with Epilepsy—Pathophysiological Implications. Brain 116, 397–414. 10.1093/brain/116.2.397 8461973

[B18] Goldstein D. S. Bentho O. Park M. Y. Sharabi Y. (2011). Low-frequency power of heart rate variability is not a measure of cardiac sympathetic tone but may be a measure of modulation of cardiac autonomic outflows by baroreflexes. Exp. Physiol. 96, 1255–1261. 10.1113/expphysiol.2010.056259 21890520 PMC3224799

[B19] Harper R. M. Bandler R. Spriggs D. Alger J. R. (2000). Lateralized and widespread brain activation during transient blood pressure elevation revealed by magnetic resonance imaging. J. Comp. Neurol. 417, 195–204. 10.1002/(SICI)1096-9861(20000207)417:2<195::AID-CNE5>3.0.CO;2-V 10660897

[B20] Heims H. C. Critchley H. D. Martin N. H. Jäger H. R. Mathias C. J. Cipolotti L.. (2006). Cognitive functioning in orthostatic hypotension due to pure autonomic failure. Clin. Auton. Res. 16, 113–120. 10.1007/s10286-006-0318-7 16683070

[B21] Imholz B. P. Dambrink J. H. Karemaker J. M. Wieling W. (1990). Orthostatic circulatory control in the elderly evaluated by non-invasive continuous blood pressure measurement. Clin. Sci. (Lond.) 79, 73–79. 2167794 10.1042/cs0790073

[B22] Janig W. Habler H. J. (2003). Neurophysiological analysis of target-related sympathetic pathways–from animal to human: similarities and differences. Acta Physiol. Scand. 177, 255–274. 10.1046/j.1365-201X.2003.01088.x 12608996

[B23] Kalisch R. Wiech K. Critchley H. D. Seymour B. O'Doherty J. P. Oakley D. A.. (2005). Anxiety reduction through detachment: subjective, physiological, and neural effects. J. Cogn. Neurosci. 17, 874–883. 10.1162/0898929054021184 15969906

[B24] Katkin E. S. Wiens S. Ohman A. (2001). Nonconscious fear conditioning, visceral perception, and the development of gut feelings. Psychol. Sci. 12, 366–370. 10.1111/1467-9280.00368 11554668

[B25] Kaye J. M. Young T. M. Mathias C. J. Watson L. Lightman S. L. (2006). Neuroendocrine and behavioural responses to CO2 inhalation in central versus peripheral autonomic failure. Clin. Auton. Res. 16, 121–129. 10.1007/s10286-006-0331-x 16475017

[B26] Khurana R. K. Setty A. (1996). The value of the isometric hand-grip test–studies in various autonomic disorders. Clin. Auton. Res. 6, 211–218. 10.1007/BF02291136 8902317

[B27] Kimmerly D. S. O'Leary D. D. Menon R. S. Gati J. S. Shoemaker J. K. (2005). Cortical regions associated with autonomic cardiovascular regulation during lower body negative pressure in humans. J. Physiol. 569, 331–345. 10.1113/jphysiol.2005.091637 16150800 PMC1464214

[B28] King A. B. Menon R. S. Hachinski V. Cechetto D. F. (1999). Human forebrain activation by visceral stimuli. J. Comp. Neurol. 413, 572–582. 10495443

[B29] Lang P. J. Bradley M. M. Cuthbert B. N. (2005). International Affective Picture System (IAPS): Affective Ratings of Pictures and Instruction Manual. Technical Report. Gainesville, FL: University of Florida.

[B30] Lee W. E. Kwok C. H. Hunter E. C. Richards M. David A. S. (2012). Prevalence and childhood antecedents of depersonalization syndrome in a UK birth cohort. Soc. Psychiatry Psychiatr. Epidemiol. 47, 253–261. 10.1007/s00127-010-0327-7 21181112 PMC3355298

[B31] Lemche E. Anilkumar A. Giampietro V. P. Brammer M. J. Surguladze S. A. Lawrence N. S.. (2008). Cerebral and autonomic responses to emotional facial expressions in depersonalisation disorder. Br. J. Psychiatry 193, 222–228. 10.1192/bjp.bp.107.044263 18757982

[B32] Lemche E. Brammer M. J. David A. S. Surguladze S. A. Phillips M. L. Sierra M.. (2013). Interoceptive-reflective regions differentiate alexithymia traits in depersonalization disorder. Psychiatry Res. 214, 66–72. 10.1016/j.pscychresns.2013.05.006 23932225 PMC4024664

[B33] Lemche E. Surguladze S. A. Giampietro V. P. Anilkumar A. Brammer M. J. Sierra M.. (2007). Limbic and prefrontal responses to facial emotion expressions in depersonalization. Neuroreport 18, 473–477. 10.1097/WNR.0b013e328057deb3 17496806

[B34] Lewin J. S. Friedman L. Wu D. Miller D. A. Thompson L. A. Klein S. K.. (1996). Cortical localization of human sustained attention: detection with functional MR using a visual vigilance paradigm. J. Comput. Assist. Tomogr. 20, 695–701. 10.1097/00004728-199609000-00002 8797896

[B35] Malliani A. Pagani M. Lombardi F. Cerutti S. (1991). Cardiovascular neural regulation explored in the frequency domain. Circulation 84, 482–492. 10.1161/01.CIR.84.2.482 1860193

[B36] Mathias C. J. (2003). Autonomic diseases: clinical features and laboratory evaluation. J. Neurol. Neurosurg. Psychiatry 74 Suppl 3, iii31–iii41. 10.1136/jnnp.74.suppl_3.iii31 12933912 PMC1765633

[B37] Mathias C. J. Iodice V. Low D. A. Bannister R. (2013). Investigation of autonomic disorders, in Autonomic Failure: A Textbook of Clinical Disorders of the Autonomic Nervous System, eds Mathias C. J. Bannister R. (Oxford: Oxford University Press), 259–289.

[B39] Mayer-Gross W. (1935). On Depersonalization. Br. J. Med. Psychol. 15, 103–126 10.1111/j.2044-8341.1935.tb01140.x

[B40] Medford N. Brierley B. Brammer M. Bullmore E. T. David A. S. Phillips M. L.. (2006). Emotional memory in depersonalization disorder: a functional MRI study. Psychiatry Res. 148, 93–102. 10.1016/j.pscychresns.2006.05.007 17085021

[B41] Moak J. P. Goldstein D. S. Eldadah B. A. Saleem A. Holmes C. Pechnik S.. (2007). Supine low-frequency power of heart rate variability reflects baroreflex function, not cardiac sympathetic innervation. Heart Rhythm 4, 1523–1529. 10.1016/j.hrthm.2007.07.019 17997358 PMC2204059

[B43] Neff R. A. Wang J. Baxi S. Evans C. Mendelowitz D. (2003). Respiratory sinus arrhythmia: endogenous activation of nicotinic receptors mediates respiratory modulation of brainstem cardioinhibitory parasympathetic neurons. Circ. Res. 93, 565–572. 10.1161/01.RES.0000090361.45027.5B 12907666

[B44] Nicotra A. Critchley H. D. Mathias C. J. Dolan R. J. (2006). Emotional and autonomic consequences of spinal cord injury explored using functional brain imaging. Brain 129, 718–728. 10.1093/brain/awh699 16330503 PMC2633768

[B45] Ongur D. Ferry A. T. Price J. L. (2003). Architectonic subdivision of the human orbital and medial prefrontal cortex. J. Comp. Neurol. 460, 425–449. 10.1002/cne.10609 12692859

[B46] Oppenheimer S. M. Gelb A. Girvin J. P. Hachinski V. C. (1992). Cardiovascular effects of human insular cortex stimulation. Neurology 42, 1727–1732. 10.1212/WNL.42.9.1727 1513461

[B47] Oppenheimer S. M. Kedem G. Martin W. M. (1996). Left-insular cortex lesions perturb cardiac autonomic tone in humans. Clin. Auton. Res. 6, 131–140. 10.1007/BF02281899 8832121

[B48] Parati G. Mancia G. di Rienzo M. Castiglioni P. (2006). Point: cardiovascular variability is/is not an index of autonomic control of circulation. J. Appl. Physiol. 101, 676–678 discussion: 681–682. 10.1152/japplphysiol.00446.2006 16645191

[B49] Pavlov I. P. (1927). Conditioned Reflexes. Oxford: Oxford University Press.

[B50] Phillips M. L. Medford N. Senior C. Bullmore E. T. Suckling J. Brammer M. J.. (2001). Depersonalization disorder: thinking without feeling. Psychiatry Res. 108, 145–160. 10.1016/S0925-4927(01)00119-6 11756013

[B51] Pollatos O. Fustos J. Critchley H. D. (2012). On the generalised embodiment of pain: how interoceptive sensitivity modulates cutaneous pain perception. Pain 153, 1680–1686. 10.1016/j.pain.2012.04.030 22658270

[B52] Rahman F. Pechnik S. Gross D. Sewell L. Goldstein D. S. (2011). Low frequency power of heart rate variability reflects baroreflex function, not cardiac sympathetic innervation. Clin. Auton. Res. 21, 133–141. 10.1007/s10286-010-0098-y 21279414 PMC3094491

[B53] Rainville P. Bechara A. Naqvi N. Damasio A. R. (2006). Basic emotions are associated with distinct patterns of cardiorespiratory activity. Int. J. Psychophysiol. 61, 5–18. 10.1016/j.ijpsycho.2005.10.024 16439033

[B54] Saper C. B. (2002). The central autonomic nervous system: conscious visceral perception and autonomic pattern generation. Annu. Rev. Neurosci. 25, 433–469. 10.1146/annurev.neuro.25.032502.111311 12052916

[B55] Schoenberg P. L. Sierra M. David A. S. (2012). Psychophysiological investigations in depersonalization disorder and effects of electrodermal biofeedback. J. Trauma Dissociation 13, 311–329. 10.1080/15299732.2011.606742 22545565

[B56] Sierra M. Baker D. Medford N. David A. S. (2005). Unpacking the depersonalization syndrome: an exploratory factor analysis on the Cambridge Depersonalization Scale. Psychol. Med. 35, 1523–1532. 10.1017/S0033291705005325 16164776

[B57] Sierra M. Berrios G. E. (2000). The Cambridge Depersonalization Scale: a new instrument for the measurement of depersonalization. Psychiatry Res. 93, 153–164. 10.1016/S0165-1781(00)00100-1 10725532

[B58] Sierra M. David A. S. (2011). Depersonalization: a selective impairment of self-awareness. Conscious Cogn. 20, 99–108. 10.1016/j.concog.2010.10.018 21087873

[B59] Sierra M. Lopera F. Lambert M. V. Phillips M. L. David A. S. (2002a). Separating depersonalisation and derealisation: the relevance of the “lesion method.” J. Neurol. Neurosurg. Psychiatry 72, 530–532. 10.1136/jnnp.72.4.530 11909918 PMC1737835

[B60] Sierra M. Medford N. Wyatt G. David A. S. (2012). Depersonalization disorder and anxiety: a special relationship? Psychiatry Res. 197, 123–127. 10.1016/j.psychres.2011.12.017 22414660

[B62] Sierra M. Nestler S. Jay E.-L. Ecker C. Feng Y. David A. S. (2014). A structural MRI study of cortical thickness in depersonalisation disorder. Psychiatry Res. 224, 1–7. 10.1016/j.pscychresns.2014.06.007 25089021

[B61] Sierra M. Senior C. Dalton J. McDonough M. Bond A. Phillips M. L.. (2002b). Autonomic response in depersonalization disorder. Arch. Gen. Psychiatry 59, 833–838. 10.1001/archpsyc.59.9.833 12215083

[B63] Simeon D. Guralnik O. Knutelska M. Yehuda R. Schmeidler J. (2003a). Basal norepinephrine in depersonalization disorder. Psychiatry Res. 121, 93–97. 10.1016/S0165-1781(03)00205-1 14572626

[B64] Simeon D. Knutelska M. Nelson D. Guralnik O. Schmeidler J. (2003b). Examination of the pathological dissociation taxon in depersonalization disorder. J. Nerv. Ment. Dis. 191, 738–744. 10.1097/01.nmd.0000095126.21206.3e 14614341

[B65] Simeon D. Kozin D. S. Segal K. Lerch B. Dujour R. Giesbrecht T. (2008). De-constructing depersonalization: further evidence for symptom clusters. Psychiatry Res. 157, 303–306. 10.1016/j.psychres.2007.07.007 17959254

[B66] Struthers A. D. Pai S. Seidelin P. H. Coutie W. J. Morton J. J. (1987). Evidence in humans for a postsynaptic interaction between noradrenaline and angiotensin II with regard to systolic but not diastolic blood pressure. J. Hypertens. 5, 671–676. 10.1097/00004872-198712000-00006 3429867

[B67] Tabibnia G. Satpute A. B. Lieberman M. D. (2008). The sunny side of fairness: preference for fairness activates reward circuitry (and disregarding unfairness activates self-control circuitry). Psychol Sci. 19, 339–347. 10.1111/j.1467-9280.2008.02091.x 18399886

[B68] Task Force of the European Society of Cardiology and the North American Society of Pacing and Electrophysiology. (1996). Heart rate variability. Standards of measurement, physiological interpretation and clinical use. Circulation 93, 1043–1065. 10.1161/01.CIR.93.5.1043 8598068

[B69] Turpin G. (1986). Effects of stimulus intensity on autonomic responding: the problem of differentiating orienting and defense reflexes. Psychophysiology 23, 1–14. 10.1111/j.1469-8986.1986.tb00583.x 3511488

[B70] West S. G. Brownley K. A. Light K. C. (1998). Postexercise vasodilatation reduces diastolic blood pressure responses to stress. Ann. Behav. Med. 20, 77–83. 10.1007/BF02884452 9989312

[B71] Westerhaus M. J. Loewy A. D. (2001). Central representation of the sympathetic nervous system in the cerebral cortex. Brain Res. 903, 117–127. 10.1016/S0006-8993(01)02453-2 11382395

[B72] Wing J. K. Cooper J. E. Sartorius N. (1974). The Description and Classification of Psychiatric Symptoms. An Instruction Manual for the PSE and CATEGO Systems. Cambridge: Cambridge University Press.

[B73] World Medical Association. (1994). Code of Ethics: declaration of Helsinki. Br. Med. J. 302, 1194.

